# Daily estimates of clinical severity of symptoms in bipolar disorder from smartphone-based self-assessments

**DOI:** 10.1038/s41398-020-00867-6

**Published:** 2020-06-18

**Authors:** Jonas Busk, Maria Faurholt-Jepsen, Mads Frost, Jakob E. Bardram, Lars Vedel Kessing, Ole Winther

**Affiliations:** 1 Department of Applied Mathematics and Computer Science, Technical University of Denmark, Lyngby, Denmark; 2 Department of Health Technology, Technical University of Denmark, Lyngby, Denmark; 3 Copenhagen Affective Disorder Research Center (CADIC), Psychiatric Center Copenhagen, Rigshospitalet, Copenhagen, Denmark; 4 Monsenso ApS, Copenhagen, Denmark; 5 Faculty of Health and Medical Sciences, University of Copenhagen, Copenhagen, Denmark; 6 Center for Genomic Medicine, Rigshospitalet, Copenhagen University Hospital, Copenhagen, Denmark; 7 Bioinformatics Centre, Department of Biology, University of Copenhagen, Copenhagen, Denmark

**Keywords:** Bipolar disorder, Depression

## Abstract

Currently, the golden standard for assessing the severity of depressive and manic symptoms in patients with bipolar disorder (BD) is clinical evaluations using validated rating scales such as the Hamilton Depression Rating Scale 17-items (HDRS) and the Young Mania Rating Scale (YMRS). Frequent automatic estimation of symptom severity could potentially help support monitoring of illness activity and allow for early treatment intervention between outpatient visits. The present study aimed (1) to assess the feasibility of producing daily estimates of clinical rating scores based on smartphone-based self-assessments of symptoms collected from a group of patients with BD; (2) to demonstrate how these estimates can be utilized to compute individual daily risk of relapse scores. Based on a total of 280 clinical ratings collected from 84 patients with BD along with daily smartphone-based self-assessments, we applied a hierarchical Bayesian modelling approach capable of providing individual estimates while learning characteristics of the patient population. The proposed method was compared to common baseline methods. The model concerning depression severity achieved a mean predicted *R*^2^ of 0.57 (SD = 0.10) and RMSE of 3.85 (SD = 0.47) on the HDRS, while the model concerning mania severity achieved a mean predicted *R*^2^ of 0.16 (SD = 0.25) and RMSE of 3.68 (SD = 0.54) on the YMRS. In both cases, smartphone-based self-reported mood was the most important predictor variable. The present study shows that daily smartphone-based self-assessments can be utilized to automatically estimate clinical ratings of severity of depression and mania in patients with BD and assist in identifying individuals with high risk of relapse.

## Introduction

Bipolar disorder (BD) is a common and complex illness with an estimated prevalence of 1–2% and is regarded as one of the most important causes of disability worldwide^1,2^. BD is characterized by recurrent episodes of depression, (hypo)mania and mixed episodes intervened by periods of euthymia^3^ and with a high degree of comorbidity and functional impairment^4^. BD is associated with an elevated risk of mortality due to suicide and medical comorbidities such as cardiovascular disease and diabetes^5–7^, and among people with BD, life expectancy is decreased 8–12 years^8,9^. In clinical practice, there are major challenges in diagnosing and treating BD^10^. Patients with BD are often misdiagnosed, and the correct diagnosis can be delayed for several years after illness onset^11–13^. Currently, due to the lack of objective tests, the diagnostic process and the clinical assessment of the severity of depressive and manic symptoms relies on subjective information, clinical evaluation and rating scales^14^. Periodic clinical evaluations using clinical rating scales such as the Hamilton Depression Rating Scale (HDRS)^15^ and the Young Mania Rating Scale (YMRS)^16^ are currently used as the golden standard for assessing the severity of depressive and manic symptoms in patients with BD. Each rating scale consists of a series of items reflecting various symptoms of depression and mania, and these items are finally added up to produce a total score summarizing the current severity of depressive (HDRS) or manic (YMRS) state of the patient. However, the use of clinical rating scales involves a risk of potential patient recall bias, other recall distortions, decreased illness insight (mainly during affective episodes) and individual clinician observer bias^17–21^. In addition, the clinical evaluations are time consuming and require a specialist who is trained and experienced in using the rating scales to produce consistent, valid and reliable results.

As part of treatment, patients may be asked to perform daily self-assessments to track changes in symptoms between clinical evaluations. Modern smartphones provide a unique platform for fine-grained real-time symptom monitoring and management, and a convenient means of self-assessment that have traditionally been carried out on paper^22–24^. A smartphone-based monitoring system enables users to ubiquitously record and review their own data, receive reminders, and even share data with carers and clinicians. From the perspective of health care providers, it offers efficient, online monitoring of a group of patients and enables intervention in case any deterioration is observed. Electronic self-monitoring has the additional benefit of making data available for immediate and automatic analysis that can help support monitoring and treatment tasks between outpatient visits.

Correlations between smartphone-based self-reported mood scores and clinical ratings of depressive and manic symptoms measured using the HDRS and the YMRS in patients with BD have already been demonstrated by previous work^25–27^, but to our knowledge this is the first study to predict scores of clinical ratings directly from combinations of smartphone-based self-assessed data in patients with BD. In related work, detection of daily self-reported mood from smartphone sensor and usage data is well studied^23,28–30^, but remains a difficult problem due to noisy data. In ref. ^31^, Grünerbl et al. classified affective states and state changes derived from clinical ratings and phone interviews of patients with BD from a combination of smartphone sensor modalities and argued that detecting deviations from the euthymic state is more important than the recognition of a particular affective state in practical applications.

Several studies in the field of affective computing have highlighted the need for personalized models to account for individual differences in order to achieve good predictive performance^29,30,32,33^. However, a separate analysis is not feasible until sufficient data about each individual is available. Hierarchical Bayesian modelling is a well-suited approach for providing individual models while borrowing statistical power from the population, which is especially useful when the individual datasets are too small to be analysed separately^34^.

The main of objective of this study was to examine the feasibility of producing daily estimates of clinical ratings of depression and mania based on smartphone self-assessments of symptoms collected from a group of patients with BD, who were followed as part of a randomized controlled trial (RCT)^35^. Additionally, we aimed to demonstrate how uncertainty in the estimated quantities could be used to compute individual, daily risk of relapse, useful for identifying high-risk individuals who need urgent assistance. Our assumption was that daily, automatic estimates of clinical ratings augmented with individual relapse risk scores are more interpretable and actionable results than observing the smartphone-based self-assessments directly and can be a valuable tool in continuous monitoring of illness activity and treatment of patients with BD.

## Materials and methods

### Patients and study design

Data analysed in this study was collected between September 2014 and January 2018 during the MONARCA II RCT, investigating the effect of smartphone-based monitoring in patients with BD^35^. All patients with a diagnosis of BD who had previously been treated at the Copenhagen Clinic for Affective Disorder, Denmark, in the period from 2004 to January 2016 and who at the time of recruitment were being treated at community psychiatric centres, private psychiatrists and general practitioners were invited to participate in the trial. The clinic is a specialized outpatient clinic with a catchment area consisting of the Capital Region in Denmark corresponding to 1.4 million people. Patients with a newly diagnosis of BD or with treatment-resistant BD were referred to the clinic. The staff consists of specialists in psychiatry, psychologists, nurses, and a social worker, all with specific experience and knowledge regarding BD. Treatment at the clinic comprises a two-year program including combined evidence-based psychopharmacological treatment and supporting therapy, including group psychoeducation^36^. Patients were included in the study for a nine-month follow-up period if they had a BD diagnosis according to ICD-10 using the Schedules for Clinical Assessments in Neuropsychiatry (SCAN)^37^ and previously were treated at the Copenhagen Clinic for Affective Disorder. Patients with schizophrenia, schizotypal or delusional disorders, previous use of the MONARCA system, pregnancy and lack of Danish language skills were excluded. Patients with other comorbid psychiatric disorders and substance use were eligible for the trial. As part of the MONARCA II trial, patients were randomized to either using a smartphone-based monitoring system (the Monsenso system) for daily self-monitoring (the intervention group) or to treatment as usual (the control group). Patients from the intervention group who successfully provided smartphone-based self-monitoring data were included in the analyses in the present study.

### Data description

#### Clinical assessments

The dataset consists of 280 clinical ratings collected from 84 patients with BD. Each clinical rating includes ratings for severity of depression and mania using the HDRS^15^ and the YMRS^16^, respectively. Each participant was evaluated by a clinician up to 5 times during the study period (at baseline, after 4 weeks, 3 months, 6 months and 9 months). All clinical assessments were conducted by a researcher (MFJ), who was blinded to all smartphone-based data. Thus, data on the severity of depressive and manic symptoms were collected rater-blinded. On both rating scales, the first item indicates mood and low severity ratings indicate low levels of either depressive or manic symptoms while high severity ratings indicate severe symptoms. A score of 13 or more on either rating scale was classified as a depressive or manic episode, respectively, while a high score on both scales at the same time constituted a mixed episode. The cut-off on the HDRS and the YMRS of 13, in contrast to a lower cut-off, was chosen á priori to increase the validity of a current affective depressive or manic/mixed state (the more severe, the higher the validity). A euthymic state was defined as HDRS and YMRS less than 13 thereby also including affective states with partial remission. Clinical ratings with the HDRS and the YMRS were considered to be valid on the day of the assessment as well as the 3 previous days, thus each rating is attributed a total of 4 days in the present dataset.

#### Smartphone-based self-assessments

In addition to periodic clinical ratings, patients were instructed to carry out daily self-assessments via a smartphone application (the Monsenso system) configured for the present study. The smartphone application was developed using an iterative, user-centred design process involving patients, IT researchers, clinicians and clinical researchers, and the items chosen for the self-assessments were designed to capture clinically important symptoms of bipolar disorder^23^. The self-assessment included the following items: activity level (scored from −3 to +3); alcohol consumption (number of units from 0 to 10+); anxiety level (scored from 0 to 2); irritability level (scored from 0 to 2); cognitive problems (scored from 0 to 2); medicine adherence (not taken/taken/taken with changes); mixed mood (yes/no); mood (scored from −3 to +3 including −0.5 and +0.5); sleep duration (in hours); and stress level (scored from 0 to 2). The activity, medicine, mood and sleep items were mandatory items, which the patients evaluated daily. Additionally, the smartphone application enabled users to configure reminders and users were allowed to provide self-assessments retrospectively for up to 2 days in case they forgot the daily entry. The entered self-assessed data collected over time was visually presented to the users on their smartphone.

### Statistical analysis

#### Data preprocessing

Three smartphone-based self-assessment variables, *mood*, *sleep* and *medicine*, required preprocessing prior to analysis. We split the mood variable into a negative and positive component, *mood negative* and *mood positive*, allowing for non-linear relationships with the clinical ratings as we expected negative mood to be associated mainly with severity of depression (reflected by scores on the HDRS) and positive mood to be associated mainly with severity of mania (reflected by scores on the YMRS). Additionally, we expected the relationship between sleep duration and symptom severity to be non-linear as increased or decreased sleep duration can both represent signs of deterioration during depression and mania. To encode this, we subtracted the individual-level mean of the sleep duration variable and split the result into positive and negative components, *sleep negative* and *sleep positive*. When testing the out-of-sample predictive performance of statistical models, the individual mean sleep duration was computed on the training set and applied to generate features in the training set and test set. The medicine adherence variable was categorical by design with categories: *medicine not taken*, *medicine taken as prescribed*, *medicine taken with changes*. To prepare the data for analysis, the three possible answers were encoded with two exclusive binary variables indicating if medicine was not taken, *medicine omitted*, or if medicine was taken with changes, *medicine changed*. The expected most common answer, *medicine taken as prescribed*, was not encoded to avoid collinearity in the regression models (a.k.a. “the dummy variable trap”). Finally, all variables were normalized by their allowed minimum and maximum values to allow for easier selection of model hyperparameters and interpretation of the inferred model weights.

It was a common problem for patients to occasionally forget to fill in their daily self-assessment, resulting in missing values in the dataset. In most cases, self-assessments were either complete for all items or missing, but in a few instances, they were only partially answered. To avoid discarding observations with only a few missing values, we experimented with filling in values from the previous day, which is a common method for dealing with missing values in time series data^38^. However, it resulted in very few additional complete observations and we therefore decided to leave this step out.

#### Modelling approach

When analysing several related sets of measurements, such as data from individuals of a population, the two extreme approaches are to either pool the datasets in a one-size-fits-all solution or to analyse the datasets separately, the latter only being possible when sufficient data is available (also known as the cold start problem). A hierarchical Bayesian approach provides an intermediate solution that enables personalized models while learning the characteristics of the population^39^. In a hierarchical Bayesian regression model, individuals have their own set of regression intercept and weights, *α*_*j*_,*β*_*j*_, sampled from a common population distribution parameterized by population-level means *μ* and variances *τ* determining the amount of pooling:

$$\begin{array}{l}\alpha _j,\beta _j\left. \sim \right.{\mathrm{Normal}}\left( {\mu ,\tau } \right)\\ y_{ji}\left. \sim \right.{\mathrm{Normal}}\left( {\alpha _j + \beta _j^T{\boldsymbol{x}}_{ji},\sigma } \right),\end{array}$$

where *y*_*ji*_ is the *i*th observation of the target variable for individual *j*, *x*_*ji*_ are the corresponding predictor variables and *σ* is the standard error. This hierarchical tying together of parameters means that data from the population helps regularize the individual-level weights. An additional benefit of the Bayesian approach is that it expresses uncertainty in all the model parameters and predictions by their posterior distributions, which is important for interpretability of the model. For further details, a complete description of the hierarchical Bayesian model is provided in the Supplementary Information (SI).

In the present study, we used Stan^40^ to specify and perform inference in the Bayesian models and then compared the predictive results with pooled and separate naïve mean baselines and common machine learning methods: Ridge Regression from the scikit-learn machine learning library^41^ and XGBoost regression from the XGBoost Python package^42^. Details of the Stan setup is also included in the SI. To estimate the predictive performance of the models we designed a cross-validation experiment where in each iteration we held out one randomly sampled clinical evaluation (consisting of up to 4 days of data) from each individual and used the remaining data to fit the models. This procedure was repeated *K* times and the predicted coefficient of determination (*R*^2^) and root mean square error (RMSE) was computed on the held-out data in each iteration. We evaluated the models on the HDRS and the YMRS total scores as well as item 1 of each rating scale, since these items reflect mood only. Additionally, we evaluated the models using all smartphone-based self-assessment items, the mandatory self-assessment items (activity, medicine, mood and sleep) and using only the mood self-assessment item, respectively. Estimating scores on the HDRS and the YMRS with separate models enables prediction of high values of the HDRS and the YMRS at the same time, indicating a mixed episode.

#### Computing risk of relapse

In some practical applications, it may be more relevant to accurately identify high-risk individuals than to estimate the exact value of the severity score. Applying a Bayesian approach does not only provide a point estimate of the outcome of interest but provides a probability distribution of unobserved (future) outcomes given previously observed data, i.e. the posterior predictive distribution, which can be utilized to reason about uncertainty in the predictions. Specifically, samples from the posterior predictive distribution can be used to compute the probability that an unobserved outcome, $\tilde y_{ji}$, exceeds a predefined threshold, *T*:

$${\mathrm{Pr}}\left( {\tilde y_{ji} \ge T} \right).$$

When estimating scores of clinical ratings, by applying a threshold *T* = 13 we can interpret this probability as the risk that an individual is experiencing severe symptoms and utilize it as a personal score indicating the risk of relapse.

### Ethical considerations

The MONARCA II RCT was approved by the Regional Ethics Committee in the Capital Region of Denmark (H-2-2014-059) and the Danish Data protection agency (2013-41-1710). The law on handling of personal data was respected. All potential participants were given both written and oral information about the study before informed consent was obtained. Prior to commencement the trial was registered at ClinicalTrials.gov (NCT02221336). Electronic data collected from the smartphones were stored at a secure server at Concern IT, Capital Region, Denmark (I-suite number RHP-292 2011-03). The trial complied with the Helsinki Declaration of 1975, as revised in 2008.

## Results

### Descriptive statistics

The MONARCA II dataset consists of 280 clinical evaluations, with a mean number of clinical evaluations per patients during the study of 3.33 (SD = 1.14), and a total of 15975 daily smartphone-based self-assessments with a mean number of smartphone-based self-assessments during the study of 190.18 (SD = 70.97) from 84 patients with BD assigned to the intervention group of the RCT. The age ranged from 21 to 71 years (mean = 43.1, SD = 12.4) and 61.9% (*N* = 52) were women. During the study period, most patients presented with rather low severity of depressive and manic symptoms resulting in low HDRS and YMRS scores. The mean HDRS total score was 7.56 (SD = 6.29) and 20.4% of scores were greater than or equal to 13. The mean YMRS total score was 2.85 (SD = 4.17) and 5.0% of scores were greater than or equal to 13. The mean HDRS item 1 score was 0.69 (SD = 0.85) and the mean YMRS item 1 score was 0.24 (SD = 0.53). Similarly, the majority of the smartphone-based self-reported mood scores were close to zero with a mean of −0.14 (SD = 0.48), indicating neutral mood (euthymia).

**Fig. 1:**
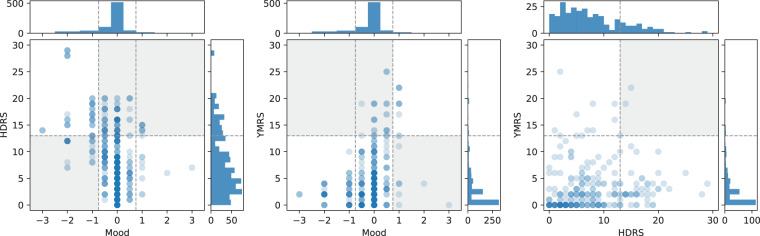
Distributions of clinical ratings of symptom severity of depression (HDRS) and mania (YMRS) and smartphone-based self-reported mood scores. A negative mood score is expected to indicate a high HDRS score and a positive mood score is expected to indicate a high YMRS score. The HDRS and YMRS scores are rarely high at the same time (indicating mixed mood). Thus, data is expected to primarily occupy the white background areas of the scatter plots.

After filling back the clinical severity ratings 4 days (since the clinical rating scales reflect this time period) there were 764 observations with associated smartphone-based self-assessments. Figure 1 shows the association between the clinical ratings and the smartphone-based self-reported mood scores. Overall, a high score on the HDRS corresponded to neutral or depressed smartphone-based self-assessed mood (*r* = −0.40, *P* < 0.01) while a high score on the YMRS corresponded to neutral or elevated smartphone-based self-assessed mood (*r* = 0.22, *P* < 0.001). Only in a few instances were the HDRS and the YMRS rated high at the same time, indicating a mixed episode (*r* = 0.13, *P* = 0.02).

### Model estimates

The hierarchical Bayesian regression model was evaluated on the entire dataset of clinical ratings combined with all self-assessed items of the completed smartphone-based self-assessments for all participants with at least two data points (*N* = 433). The model predicting total scores on the HDRS achieved an *R*^2^ of 0.84, indicating that the model accounted for 84% of the variance in the data, and a residual RMSE of 2.41. The model predicting total scores on the YMRS achieved an *R*^2^ of 0.81 and a residual RMSE of 2.07. The model predicting the HDRS item 1 score achieved an *R*^2^ of 0.89 and a residual RMSE of 0.30, and the model predicting the YMRS item 1 score achieved an *R*^2^ of 0.86 and a residual RMSE of 0.22.

The distributions of inferred population-level mean, *μ*, and variance, *τ*, parameters in the hierarchical Bayesian regression HDRS total and YMRS total models are summarized in Table 1. The absolute *t*-statistic of the mean parameters, computed as the mean scaled by the standard error of the parameter: $t_\mu = \bar \mu /{\it{SE}}(\mu )$, is included as a measure of variable importance, following the intuition that larger absolute weights and lower variance implies importance^43^. This shows that negative mood was the most important predictor variable in the HDRS model while positive mood was the most important predictor and in the YMRS model. A visual presentation of the population-level parameters and a weight matrix summarising the individual parameters are included in the SI. A figure showing the effect size of each self-assessment item is also included in the SI.

**Table 1:** Weight table showing the population-level parameters in the HDRS total model (top) and the YMRS total model (bottom).

HDRS							
	*μ*^a^				*τ*^b^		
Predictor	Mean (SD)	95% CI^c^		|*t*|^d^	Mean (*SD*)	95% CI^c^	
Intercept	6.43 (0.66)	5.13	7.73	9.67	4.10 (0.50)	3.23	5.19
Mood negative	−9.11 (1.40)	−11.94	−6.43	6.51	0.56 (0.40)	0.02	1.50
Sleep negative	−6.48 (1.66)	−9.72	−3.19	3.89	0.42 (0.31)	0.02	1.16
Mixed Mood	2.11 (0.67)	0.79	3.42	3.15	0.44 (0.32)	0.02	1.20
Anxiety	2.26 (0.86)	0.58	3.96	2.63	0.38 (0.28)	0.02	1.06
Medicine changed	−1.81 (0.71)	−3.19	−0.40	2.55	0.35 (0.27)	0.01	0.99
Cognitive difficulty	1.09 (0.73)	−0.35	2.48	1.50	0.43 (0.32)	0.02	1.19
Mood positive	−2.80 (1.94)	−6.59	0.94	1.44	0.42 (0.32)	0.02	1.19
Sleep positive	2.83 (2.05)	−1.09	6.90	1.38	0.41 (0.31)	0.02	1.15
Activity	0.53 (0.61)	−0.66	1.71	0.88	0.50 (0.35)	0.02	1.29
Stress	0.56 (0.73)	−0.86	1.99	0.76	0.50 (0.36)	0.02	1.32
Alcohol	0.59 (1.01)	−1.39	2.54	0.59	0.41 (0.31)	0.02	1.15
Medicine omitted	0.52 (0.97)	−1.38	2.42	0.54	0.37 (0.28)	0.01	1.04
Irritable	0.05 (0.74)	−1.41	1.49	0.06	0.59 (0.42)	0.02	1.57

YMRS							
	*μ*^a^				*τ*^b^		
Predictor	Mean (SD)	95% CI^c^		|*t*|^d^	Mean (*SD*)	95% CI^c^	
Intercept	3.10 (0.68)	1.79	4.46	4.59	4.35 (0.50)	3.48	5.40
Mood positive	12.83 (1.90)	9.09	16.53	6.75	0.57 (0.42)	0.02	1.57
Mood negative	3.42 (1.30)	0.87	5.99	2.63	0.66 (0.46)	0.03	1.68
Irritable	1.31 (0.69)	−0.05	2.68	1.90	0.71 (0.47)	0.04	1.73
Mixed mood	1.02 (0.62)	−0.20	2.21	1.65	0.54 (0.36)	0.03	1.34
Stress	1.15 (0.70)	−0.21	2.54	1.65	1.24 (0.53)	0.12	2.18
Sleep positive	−2.69 (1.84)	−6.30	0.84	1.46	0.40 (0.30)	0.01	1.12
Activity	−0.78 (0.56)	−1.88	0.30	1.39	0.63 (0.41)	0.03	1.52
Medicine changed	0.46 (0.71)	−0.99	1.81	0.64	0.80 (0.48)	0.05	1.76
Cognitive difficulty	0.41 (0.69)	−0.92	1.78	0.59	0.94 (0.54)	0.05	1.99
Anxiety	0.18 (0.80)	−1.40	1.73	0.23	0.69 (0.48)	0.03	1.76
Sleep negative	0.30 (1.50)	−2.63	3.26	0.20	0.43 (0.32)	0.02	1.17
Alcohol	0.05 (0.93)	−1.77	1.86	0.06	0.39 (0.30)	0.02	1.11
Medicine omitted	−0.02 (0.90)	−1.78	1.75	0.02	0.41 (0.31)	0.01	1.15

The population-level regression weight means, *μ*, are summarized in the leftmost columns and sorted by variable importance computed as the absolute *t*-statistic of the mean parameter. The corresponding variances, *τ*, are summarized in the columns to the right and can be interpreted as the amount of pooling of the given variable in the hierarchical model.

^a^Population-level regression weight means.

^b^Population-level variance can be interpreted as the amount of pooling of the given variable in the hierarchical model.

^c^Credible interval.

^d^Absolute *t*-statistic of the mean parameter indicating variable importance.

### Cross-validation results

The predictive performance of the hierarchical Bayesian model was evaluated in *K* = 100 cross-validation experiments on all data where participants had complete observations of clinical ratings and smartphone-based self-assessments from at least three different clinical evaluations (*N* = 329). In each iteration, data from one randomly sampled clinical evaluation from each patient was held out and the remaining data was used to fit the models. Models were fitted to predict HDRS total, YMRS total, HDRS item 1 and YMRS item 1, from (1) all; (2) mandatory and (3) mood self-assessment items, respectively. The hierarchical Bayesian model was compared to naïve pooled and separate mean models along with pooled and separate ridge regression and XGBoost regression models.

Table 2 presents the cross-validation results of predicting HDRS total and YMRS total. Because of low variance in the data, the naïve mean models performed relatively well. Still the hierarchical Bayesian regression model achieved the best overall performance in every case and was significantly better than the separate mean model in both the HDRS and YMRS case according to independent *t*-tests (*P* < 0.001). Overall, the separate models performed better than their pooled counterparts. Table 3 presents the cross-validation results of predicting HDRS item 1 and YMRS item 1, indicating mood. The pooled XGBoost achieved the best result at predicting HDRS item 1 using all self-assessment items. When reducing the feature set to the mandatory or mood self-assessment items, the hierarchical Bayesian model was best. It was not possible to predict YMRS item 1 significantly better than the naïve mean baselines.

**Table 2:** Results of *K* = 100 cross-validation experiments with the HDRS total score (left columns) and the YMRS total score (right columns) models based on all, mandatory and mood self-assessment items, respectively.

	HDRS total score		YMRS total score	
Model	*R*^2^ (SD) ↑^a^	RMSE (SD) ↓^b^	*R*^2^ (SD) ↑^a^	RMSE (*SD*) ↓^b^
*All self-assessment items*				
Pooled naïve mean	−0.02 (0.03)	5.99 (0.37)	−0.04 (0.05)	4.18 (0.70)
Pooled Ridge	0.37 (0.10)	4.68 (0.48)	0.02 (0.15)	4.03 (0.60)
Pooled XGBoost	0.44 (0.10)	4.40 (0.41)	−0.04 (0.21)	4.11 (0.53)
Pooled Bayesian	0.36 (0.12)	4.72 (0.51)	0.00 (0.21)	4.04 (0.56)
Separate naïve mean	0.47 (0.11)	4.29 (0.47)	−0.00 (0.33)	4.00 (0.53)
Separate Ridge	0.47 (0.12)	4.30 (0.49)	0.04 (0.30)	3.92 (0.54)
Separate XGBoost	0.27 (0.15)	5.03 (0.49)	−0.38 (0.50)	4.64 (0.45)
Hierarchical Bayesian	**0.57 (0.10)**	**3.85 (0.47)**	**0.12 (0.31)**	**3.74 (0.46)**
*Mandatory self-assessment items*				
Pooled naïve mean	−0.02 (0.03)	5.94 (0.37)	−0.04 (0.06)	4.25 (0.71)
Pooled Ridge	0.21 (0.07)	5.24 (0.34)	0.01 (0.09)	4.12 (0.65)
Pooled XGBoost	0.37 (0.12)	4.63 (0.39)	−0.06 (0.18)	4.23 (0.57)
Pooled Bayesian	0.21 (0.10)	5.22 (0.37)	0.03 (0.13)	4.08 (0.61)
Separate naïve mean	0.46 (0.16)	4.28 (0.57)	−0.01 (0.30)	4.08 (0.54)
Separate Ridge	0.46 (0.16)	4.29 (0.57)	0.00 (0.29)	4.06 (0.54)
Separate XGBoost	0.25 (0.18)	5.06 (0.54)	−0.34 (0.39)	4.68 (0.42)
Hierarchical Bayesian	**0.54 (0.13)**	**3.94 (0.53)**	**0.10 (0.27)**	**3.85 (0.49)**
*Mood self-assessment item*				
Pooled naïve mean	−0.02 (0.02)	5.91 (0.41)	−0.05 (0.05)	4.20 (0.77)
Pooled Ridge	0.21 (0.06)	5.19 (0.35)	0.02 (0.07)	4.05 (0.70)
Pooled XGBoost	0.34 (0.11)	4.75 (0.35)	0.01 (0.18)	4.03 (0.54)
Pooled Bayesian	0.20 (0.12)	5.23 (0.45)	0.04 (0.12)	4.00 (0.63)
Separate naïve mean	0.44 (0.15)	4.31 (0.47)	0.02 (0.27)	3.98 (0.59)
Separate Ridge	0.45 (0.15)	4.29 (0.48)	0.03 (0.27)	3.96 (0.59)
Separate XGBoost	0.42 (0.15)	4.42 (0.42)	−0.04 (0.34)	4.05 (0.51)
Hierarchical Bayesian	**0.51 (0.14)**	**4.05 (0.45)**	**0.16 (0.25)**	**3.68 (0.54)**

The hierarchical Bayesian model achieved the best overall performance in every case and could predict the clinical severity ratings within 4 points of RMSE on the original rating scales. The best HDRS total result was achieved using all self-assessment items while the best YMRS total result was achieved using only the mood self-assessment item.

Bold values indicates the best results within each set of self-assessment items.

^a^Coefficient of determination. Higher is better.

^b^Root Mean Square Error. Lower is better.

**Table 3:** Results of *K* = 100 cross-validation experiments with the HDRS item 1 score (left columns) and YMRS item 1 score (right columns) models based on all, mandatory and mood self-assessment items, respectively.

	HDRS item 1 score		YMRS item 1 score	
Model	*R*^2^ (SD) ↑^a^	RMSE (SD) ↓^b^	*R*^2^ (SD) ↑^a^	RMSE (SD) ↓^b^
*All self-assessment items*				
Pooled naïve mean	−0.03 (0.04)	0.95 (0.07)	−0.05 (0.07)	0.61 (0.10)
Pooled Ridge	0.41 (0.08)	0.71 (0.06)	−0.09 (0.13)	0.62 (0.09)
Pooled XGBoost	**0.50 (0.11)**	**0.66 (0.07)**	−0.17 (0.20)	0.64 (0.09)
Pooled Bayesian	0.38 (0.14)	0.73 (0.10)	−0.16 (0.20)	0.63 (0.09)
Separate naïve mean	0.35 (0.15)	0.75 (0.08)	−0.27 (0.33)	0.66 (0.08)
Separate Ridge	0.37 (0.15)	0.73 (0.07)	−0.23 (0.30)	0.65 (0.08)
Separate XGBoost	0.18 (0.17)	0.84 (0.07)	−0.35 (0.34)	0.68 (0.06)
Hierarchical Bayesian	0.40 (0.12)	0.72 (0.06)	−0.07 (0.24)	0.61 (0.08)
*Mandatory self-assessment items*				
Pooled naïve mean	−0.03 (0.04)	0.93 (0.06)	−0.04 (0.06)	0.60 (0.08)
Pooled Ridge	0.32 (0.07)	0.75 (0.05)	0.01 (0.10)	0.59 (0.08)
Pooled XGBoost	0.39 (0.13)	0.71 (0.07)	−0.17 (0.22)	0.63 (0.08)
Pooled Bayesian	0.33 (0.13)	0.75 (0.09)	−0.03 (0.17)	0.59 (0.08)
Separate naïve mean	0.35 (0.13)	0.73 (0.08)	−0.25 (0.27)	0.65 (0.08)
Separate Ridge	0.37 (0.13)	0.72 (0.08)	−0.22 (0.25)	0.64 (0.08)
Separate XGBoost	0.14 (0.14)	0.84 (0.07)	−0.36 (0.36)	0.67 (0.06)
Hierarchical Bayesian	**0.44 (0.10)**	**0.68 (0.07)**	0.00 (0.22)	0.58 (0.08)
*Mood self-assessment item*				
Pooled naïve mean	−0.03 (0.04)	0.94 (0.07)	−0.07 (0.15)	0.61 (0.09)
Pooled Ridge	0.34 (0.07)	0.75 (0.05)	0.01 (0.16)	0.58 (0.09)
Pooled XGBoost	0.40 (0.12)	0.71 (0.07)	−0.04 (0.27)	0.59 (0.09)
Pooled Bayesian	0.33 (0.12)	0.76 (0.09)	0.02 (0.21)	0.58 (0.08)
Separate naïve mean	0.34 (0.12)	0.75 (0.07)	−0.36 (0.62)	0.66 (0.08)
Separate Ridge	0.36 (0.12)	0.74 (0.07)	−0.35 (0.61)	0.66 (0.08)
Separate XGBoost	0.37 (0.13)	0.73 (0.07)	−0.21 (0.51)	0.63 (0.08)
Hierarchical Bayesian	**0.47 (0.10)**	**0.67 (0.07)**	−0.08 (0.45)	0.59 (0.08)

The best HDRS item 1 result was achieved using the XGBoost model with all self-assessment items while YMRS item 1 could not be estimated significantly better than the naïve baseline models.

Bold values indicates the best results within each set of self-assessment items.

^a^Coefficient of determination. Higher is better.

^b^Root Mean Square Error. Lower is better.

### Predicted risk of relapse scores

**Fig. 2:**
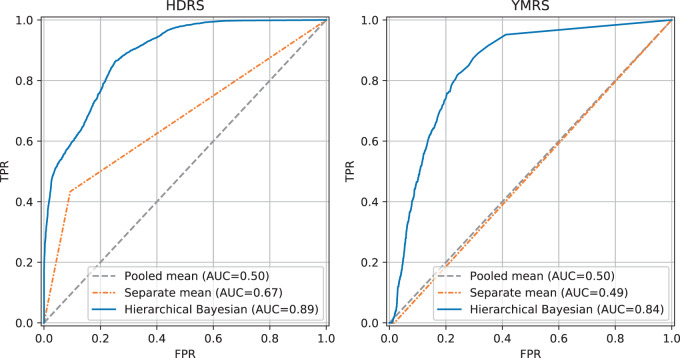
Results of predicting relapse risk scores evaluated as a binary classification problem and presented in receiver operating characteristic (ROC) curves. In both the HDRS case (left) and the YMRS case (right), the hierarchical Bayesian regression model outperforms naïve pooled and separate mean models.

The results from cross-validation experiments predicting the HDRS total score and the YMRS total score using all self-assessment items presented in the previous section were used to compute risk of relapse scores ${\mathrm{Pr}}\left( {\tilde y_{{\it{ji}}} \ge {\it{T}} = 13} \right)$. The ability of the model to correctly assign high risk to instances with high ratings can be evaluated as a binary classification problem with severity ratings equal to or greater than the threshold *T* constituting the positive class. Figure 2 presents receiver operating characteristic (ROC) curves of the HDRS total and the YMRS total models illustrating the trade-off between true positive rate (TPR) and false positive rate (FPR), comparing the hierarchical Bayesian regression model to the naïve pooled and separate mean models. The pooled mean model corresponds to a model that either classifies all instances as low risk or high risk, achieving an area under the curve (AUC) of 0.50 in both the HDRS and YMRS case. The separate mean model independently classifies each individual as either high or low risk based on observed values of the ratings and achieved an AUC of 0.67 in the HDRS case and AUC of 0.49 in the YMRS case. The hierarchical Bayesian regression model was able to account for information in the smartphone-based self-assessments as well as individual differences and achieved the highest AUC of 0.89 in the HDRS case and 0.84 in the YMRS case.

## Discussion

In the present study, we analysed clinical ratings of depression reflected by the HDRS and mania reflected by the YMRS along with daily smartphone-based self-assessments including self-reported mood in a population of 84 patients with BD. As hypothesized, there was a negative correlation between the HDRS and self-reported mood and a positive correlation between the YMRS and mood. This confirms previous work^25–27^, and suggests that smartphone-based self-reported mood is a valid indicator of symptom severity in patients with BD and thereby a clinically relevant feature for monitoring and analysis.

Interestingly and as hypothesized, the proposed approach of applying hierarchical Bayesian regression models was able to fit the data distributions of the HDRS total score and the YMRS total score and all smartphone-based self-assessment items and accounted for more than 80% of the variance in the data according to *R*^2^. Using the absolute *t*-statistic of the population-level regression weights as a measure of variable importance, decreased and increased smartphone-based self-reported mood were the most important variables for predicting the severity of depression (HDRS) and mania (YMRS). This is not surprising since sampling of self-reported mood from the patients was designed to collect indicators on the patient’s affective state and thus should reflect the clinically rated symptoms. Other important variables in the HDRS total model were decreased sleep and feelings of mixed mood and anxiety, while in the YMRS total model only mood ranked important (see Table 1).

To assess the predictive performance of the hierarchical Bayesian model compared to pooled and separate baseline models, we performed cross-validation experiments of estimating the HDRS total score, the YMRS total score, the HDRS item 1 score and the YMRS item 1 score using all smartphone-based self-assessment items, the four mandatory items and mood self-assessment item alone, respectively. Thus, we were able to estimate the total clinical rating scores using regression models based on smartphone-based self-assessments. The hierarchical Bayesian model achieved the best performance in predicting the HDRS total and was significantly better than a naïve model using the observed individual (separate) mean as a prediction (*P* < 0.001). Similarly, the hierarchical Bayesian model was best at predicting the YMRS total score and was significantly better than the naïve separate mean model. Additionally, we tested models for predicting the first item of the HDRS and the YMRS, indicating mood. The pooled XGBoost model achieved the best result in predicting the HDRS item 1 score, while estimating the YMRS item 1 score could not be improved over the naïve baseline. In all the presented experiments, we found that models based only on self-assessed mood were able to retain most of the predictive performance of models based on all self-assessment items. This further shows that mood is the most important self-reported predictor variable for estimating scores of the HDRS and the YMRS. Overall, the YMRS models did not account for much of the variance in the data, indicated by the low *R*^2^ scores. This could be mainly due to low variation in the observed YMRS data.

In clinical settings of monitoring illness activity in patients with bipolar disorder, detecting individuals with a high risk of relapse is highly important in order to enable intervention. Therefore, a sensitive indication if a symptom severity rating is above a critical threshold might be more useful than estimating the exact value of the severity rating itself. Thus, we demonstrated how uncertainty in the estimated total severity scores can be utilized to compute individual daily risk of relapse scores by considering samples from the posterior predictive distribution of the hierarchical Bayesian model. In the case of both the HDRS and the YMRS, using hierarchical Bayesian approach achieved substantial improvements over naïve models using pooled and separate means of observed data as predictions. Hence, including self-assessments in a regression model provided additional useful information for estimating the level of the clinical severity ratings and hence the relapse risk scores, which is a promising and clinically relevant result.

The findings that a combination of fine-grained daily smartphone-based self-assessment items can be used to estimate and predict clinical ratings are interesting and innovative. Daily longitudinal self-monitoring of mood symptoms gives valuable information of mood fluctuation experienced by patients with BD between clinical outpatient visits. Long-term monitoring of symptoms has been an essential part of the monitoring and treatment of BD for decades^44^ and rapidly evolving smartphone technologies have made it possible to monitor symptoms more continuously, fine-grained and in real-time. This can be clinically relevant for detection of symptoms before the first or recurrent depressive or manic episodes^45^, and allow for early intervention on prodromal symptoms. In the latest version of the Diagnostic and Statistical Manual of Mental Disorders (DSM-V), increased activity level or energy is acknowledged as a core feature of hypomania and mania together with mood changes^46^. Several studies using factor analysis have described activation and not mood state as the primary symptom in manic episodes^47,48^. However, in the present study we found mood to be the most important predictor variable for estimating the HDRS and the YMRS severity ratings while activity presented with low importance in both models. Furthermore, sleep disturbances and anxiety has been identified as early symptoms of depression and mania^49,50^, which is in line with our findings in the HDRS model while sleep and anxiety were less important in the YMRS model.

### Advantages

The patients included in the present study were clinically well characterized and were receiving treatment or had received treatment at the Copenhagen Clinic for Affective Disorders, Denmark. The clinical evaluations were conducted multiple times during follow-up by experienced researchers with a specific knowledge within BD. The smartphone-based self-assessment system used in the present studies (the Monsenso system) was developed by the authors and has been shown easy to use with a high usability, usefulness, ease of learning to use and interface quality—also when compared with other smartphone-based self-assessment systems^22,51^. The use of smartphones for fine-grained real-time monitoring reduced the risk of recall bias. The proposed hierarchical Bayesian modelling approach is well suited for analysis of small related datasets, especially when the individual datasets are too small to analyse separately. Additionally, the linear regression method and ability to express uncertainty in all estimated quantities makes the model easy to interpret, which is essential in a clinical setting. Overall, the findings from the present study are found to be innovative and generalizable to patients with BD not presenting with an acute affective episode and who are willing to use a monitoring tool during prolonged time periods.

### Limitations

The dataset used in this study primarily contained clinical ratings of low severity of affective symptoms indicating most participants did not experience severe symptoms of depression or mania during the study period. Similarly, a large proportion of the self-reported mood scores were close to zero (indicating euthymia) and had low variance. Consequently, the naïve mean baseline models could fit the data well and achieved good performance in the prediction task. However, the best regression model was still significantly better than the naïve mean models, showing that it is possible to utilize smartphone-based self-reported data to produce more accurate estimates of the clinical ratings of symptom severity. Although we saw significant correlations between self-reported mood and the HDRS and the YMRS, respectively, the correlations were weaker than what has been reported in some other studies^45^. Furthermore, the absence of high ratings makes it difficult to reason about the performance of the models in detecting extreme cases, which are the most critical in a monitoring and intervention application.

Our analysis does not explore the distribution of missing data and thus assumes data is missing at random. However, it is reasonable to believe that individuals who are experiencing severe depression or mania have difficulties coping with self-assessment while euthymic individuals find it less relevant. Thus, analysing the missing data distribution might hold valuable information regarding symptom severity which can be explored further.

Lastly, our analysis did not include any temporal information in the models, but rather used smartphone self-assessment data from a given day to estimate clinical ratings on the same day and treated each day independently from other days. Thus, the analysis made no assumptions regarding temporal patterns of mood but relied entirely on relationship between data collected on the same day.

### Perspectives and future implications

Smartphones have become a ubiquitous technology in modern society and can be utilized to provide improved and personalized illness management and monitoring in psychiatry. Smartphone-based self-assessment makes data available for immediate analysis and can enable new tools for improved illness monitoring. In particular, accurate, daily estimates of symptom severity could help identify critical cases and enable timely and individualized intervention. Additionally, advances in sensor technology and algorithms is making it possible to extract a growing range of increasingly accurate behavioural features directly from sensor data. Utilizing these automatically generated features to infer symptom severity scores could be used to eliminate the need for frequent, intrusive self-assessments and improve the user experience of illness monitoring systems in psychiatry going forward.

In this paper, we have explored the relationship between smartphone-based self-assessments and clinical ratings observed on the same day with the purpose of identifying current high-risk individuals. A related objective with possible great clinical potential would be to predict individual risk of relapse ahead of time. We see this as an important topic for future studies.

## Conclusions

In the present study, clinical ratings of the severity of depression and mania were estimated from smartphone-based self-assessments collected from patients with BD. We found that our approach of applying a hierarchical Bayesian model could estimate severity of depression and mania with low error compared to commonly used baseline methods and within 4 points of RMSE on the HDRS and the YMRS rating scales. Furthermore, we showed how uncertainty in the estimates can be utilized to compute personal relapse risk scores suited for identifying critical cases of patients experiencing severe symptoms and that our approach achieved substantial improvements over naïve pooled and separate mean models. The results presented in this work show that it is feasible to compute daily estimates of clinical severity ratings of depression and mania from smartphone-based self-assessments, which can be used to improve and automate continuous disease monitoring and treatment of BD.

## Supplemental information

Supplemental information
