# Supplementary material for: Daily estimates of clinical severity of symptoms in bipolar disorder from smartphone-based self-assessments
Source: Transl Psychiatry. 2020 Jun 18;10:194. doi: 10.1038/s41398-020-00867-6 (PMC7303106; doi:10.1038/s41398-020-00867-6)
Supplement: Supplementary file 1 — Supplemental information [file 41398_2020_867_MOESM1_ESM.pdf]

Supplementary Information

Complete hierarchical Bayesian regression model

For individual $j = 1 : J$, observation $i = 1 : N$, target variable y_{ji} and predictor variables $\mathbf{x}_{ji}$:

$$y_{ji} \sim \text{Normal}(\alpha_j + \beta_j^T \mathbf{x}_{ji}, \sigma)$$

where α_j and β_j are sampled from population distributions:

$$\alpha_j \sim \text{Normal}(\mu_\alpha, \tau_\alpha)$$
$$\beta_j \sim \text{Normal}(\mu_\beta, \tau_\beta)$$

with independent normal priors on μ, τ and σ. A Bayesian network of the hierarchical Bayesian regression model is presented in Figure SI.1.

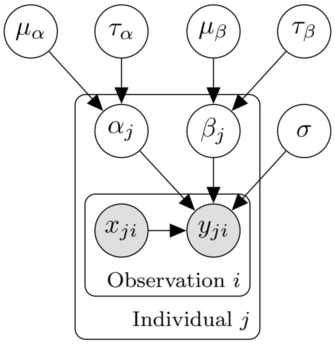

Figure SI.1: Bayesian network of a hierarchical linear regression model. Individual regression intercept α_j and weights β_j are drawn from population distributions parameterized by μ_α, τ_α and μ_β, τ_β, This allows the model to account for individual differences while constraining individual parameters to be similar across the population.

Stan details

The pooled and hierarchical Bayesian models were fitted using the statistical modeling platform Stan [40]. Stan provides tools for specifying statistical models in a probabilistic programming language and performing inference in the models. The outputs of the inference are posterior samples of model parameters along with an inferential summary. The models in this work were fitted using 4 sampling chains and 5,000 iterations where the first half was warm-up.

Distributions of the population-level regression parameters

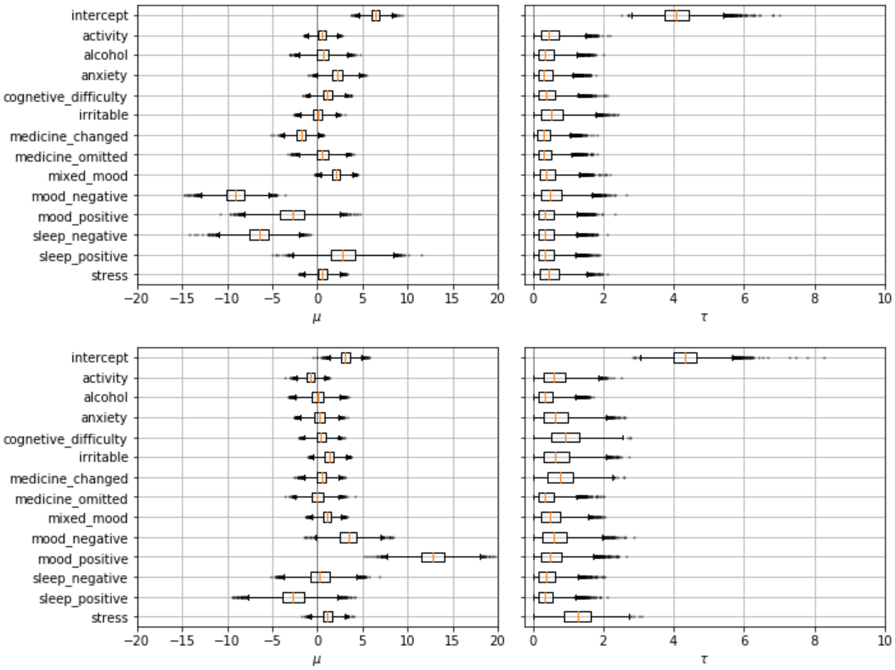

Figure SI.2: Distributions of the population-level regression parameters, μ, and variances, τ, in the hierarchical HDRS model (top) and YMRS model (bottom). In both models, self-reported mood has the largest absolute weight and group variance.

Weight matrices

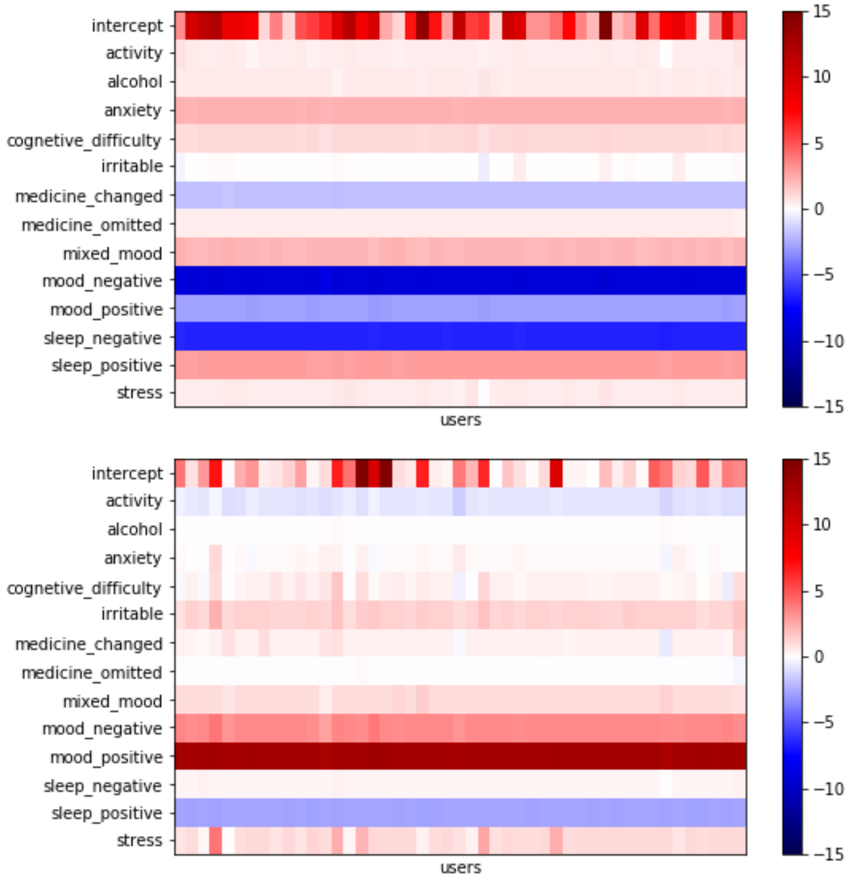

Figure SI.3: Weight matrices summarising the individual-level parameters, α, β, in the HDRS (top) and YMRS (bottom) models.

Effects

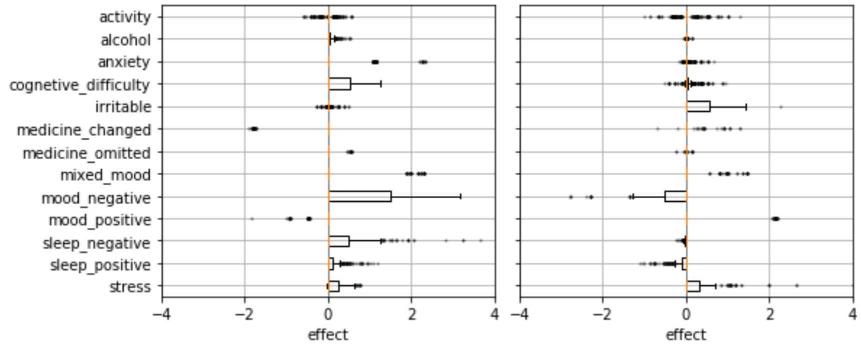

Figure SI.4: Effect plots of the HDRS model (left) and YMRS model (right) computed on the entire dataset. The effect is computed as the product between individual-level weights of the hierarchical Bayesian HDRS and YMRS models and the observed data. Because the dataset is sparse, i.e. many values are zero, the effects are often small and, consequently, substantial effects represents outliers.
